# Main factors associated with variability in the assessment of the objective response rate. A re‐analysis of the PHEREXA phase 3 clinical trial

**DOI:** 10.1002/bcp.70464

**Published:** 2026-01-31

**Authors:** Gennaro Daniele, Tommaso Giovagnoli, Pierluigi Navarra, Cinzia Dello Russo

**Affiliations:** ^1^ Phase 1 Unit, Fondazione Policlinico Universitario Agostino Gemelli IRCCS Università Cattolica del Sacro Cuore Rome Italy; ^2^ Department of Translational Medicine and Surgery, Section of Pharmacology, Fondazione Policlinico Universitario A. Gemelli IRCCS, Rome, Italy; Università Cattolica Del Sacro Cuore Rome Italy; ^3^ Department of Pharmacology & Therapeutics, Institute of Systems Molecular and Integrative Biology (ISMIB) University of Liverpool Liverpool UK

**Keywords:** BICR, blinded independent central review, inter‐reader variability, local investigators, objective response rate, RECIST

## Abstract

**Aim:**

Local investigators (LIs) overestimate the objective response rate (ORR) in comparison to Blinded Independent Central Reviewers (BICR) in oncology. In this study, we re‐analysed data obtained in the PHEREXA trial (NCT01026142) with the following aims: i) to confirm at the single‐patient level the discrepancy observed by analysing aggregated data; ii) to investigate the causes underlying such discrepancy.

**Methods:**

Individual data, available on the VIVLI platform, were analysed. For each patient, the best response was established for LIs and BICR. “Eligible” patients were those with at least one target lesion at screening. The degree of agreement was assessed through the Cohen's Kappa coefficient. Multivariate logistic regression analysis was performed to understand the reasons underlying the discrepancy.

**Results:**

Eligible subjects were 364/452 according to LIs and 371/450 per BICR. We confirmed that LIs overestimate the ORR. We found moderate agreement between the two evaluations (Cohen's κ= 0.48, p < 0.001). The probability of discrepancy increased by 20% with the increasing number of target lesions evaluated. Selection of 8–10 lesions was associated with an increased risk of discrepancy (OR, 5.27; CI 95% = 1.21–24.92; p < 0.03). The evaluation of breast, lung and lymph nodes (only) significantly increased the probability of discrepancy in comparison to the analysis of lymph nodes + organ.

**Conclusion:**

The overestimation of the tumour response by LIs increases with the number of target lesions analysed as well as with specific evaluation sites. Thus, the adoption of the two evaluations appears necessary in uncontrolled trials to provide a better estimate of tumour response.

**What is already known about this subject**

- Investigator expectation bias interferes with the analysis and interpretation of experimental results.
- In the oncology setting, the main regulatory bodies recommend the adoption of Blinded Independent Central Review (BICR) for outcomes based on tumour assessment.
- We have shown that local investigators (LIs) tend to overestimate the objective response rate (ORR) *vs*. BICR.

**What this study adds**

- We reanalysed data from the PHEREXA trial, confirming ORR overestimation by LIs
- The probability of discrepancy increased by 20% with the number of target lesions evaluated
- The evaluation of breast, lung and lymph nodes (only) significantly increased the probability of discrepancy *vs*. the analysis of lymph nodes + organ.

## 1 INTRODUCTION

The expectation bias, also referred to as the Rosenthal effect, can distort the results of a clinical trial due to the expectation of the investigators, or the patients, about the outcomes. In the field of oncology (where the expectations may be higher because of the higher unmet medical needs), the expectation bias in the patients, corresponding indeed to a placebo effect, is seldom associated with positive responses to treatments.^1^ On the other hand, when present in the investigators, expectation bias can possibly interfere with the analysis and interpretation of the experimental results. This is even more relevant in modern oncology, when numerous drugs are approved via expedited regulatory pathways, with approval often based on the results of early phase trials, i.e., trials lacking a comparator arm.^2^ Even in the advanced phases of drug development, truly blinding of treatments may be difficult, due, for example, to the different routes of drug administration or peculiar side effects among experimental groups. Therefore, an open‐label design is often chosen, increasing the possibility that expectation bias by local investigators (LIs) may affect the results.^3^ Consequently, the main regulatory bodies recommend the adoption of Blinded Independent Central Review (BICR) for all clinical outcomes based on tumour assessment, including the overall response rate (ORR) and progression‐free survival (PFS), with the potential exception for double‐blinded clinical trials by the US Food and Drug Administration (FDA).^4^ Similarly, the European Medicines Agency (EMA) states that the open‐label design of a clinical trial ‘*has implications with respect to choice of study endpoints, independent review, conduct of sensitivity analyses and other measures*’ to limit potential bias.^5^ There has been an ongoing debate on the added value of BICR to assess the efficacy of new anticancer drugs. For example, Dodd and collaborators first raised the issue of BICR as an unnecessary, expensive and time‐consuming procedure, based on the analysis of 7 trials showing no difference in the evaluation of drug efficacy between the two assessments.^6^ This was further sustained by a larger analysis carried out by a consortium of pharmaceutical companies on 27 Phase III trials with the dual evaluation of PFS. This study showed a strong correlation between the two assessments indeed.^7^

In this context, we have carried out our own analysis and previously performed a number of studies to investigate the weight of expectation bias on the outcomes of oncology trials, considering that an increasing number of papers currently report the results of both LI and BICR assessments carried out on the major endpoints susceptible to interpretation variability, i.e., PFS and ORR.^8^ In our studies, we used the following methodological approach. For each endpoint and each pair of data, a discrepancy index (DI) was calculated, i.e., the difference ‐ expressed as the ratio ‐ between the assessments carried out by LIs and BICR, respectively.^9^ Our analysis of the discrepancies between the PFS assessed by BICR and LI confirmed previous findings,^6^, ^7^ further reinforcing the notion that there is no significant difference between the assessments of PFS carried out by LIs compared to BICR.^9^, ^10^ On the other hand, we found that a significant difference exists (17.5% on average) between the assessment of ORR carried out by LIs compared to BICR in phase‐2 studies conducted in single groups of patients, with the investigators being more ‘optimistic’ than BICR in the assessment of disease state.^11^ Such a discrepancy in ORR assessment was confirmed in a subsequent analysis performed on controlled trials.^10^ Interestingly, in this study, the more optimistic assessment by LIs was equally observed in both the control and experimental arms, with the discrepancy observed in the control groups counterbalancing that of the experimental groups.^10^

At this stage, we reached a methodological limit, since no further analysis on aggregated data could probably explain the reasons behind the significant discrepancies observed between LIs and BICR in the assessment of the disease state. This point was even more challenging if one considers that such discrepancies are not observed in the assessment of PFS, which is also based on the same standardized methodologies (i.e., the response evaluation criteria in solid tumours, RECIST and analogues).^12^, ^13^ On the other hand, an analysis at the single‐patient level could address the above issues. Access to raw participant data allows to examine individual data points directly, enabling a more granular analysis of potential factors underlying discrepancies between LIs and BICR in the ORR assessment. We hypothesized that differences in the application of RECIST eligibility criteria, in the selection of target and non‐target lesions, or in the timing of events could contribute to this variability. We identified the PHEREXA trial (Clinicaltrials.gov identifier: NCT01026142) as a valuable study case to test our hypothesis for these reasons: *i*) we previously estimated an approximately 11.5% discrepancy between LIs and BICR. The DI on the ORR was 1.13 (37.2% ORR by LIs/32.9% ORR by BICR) in Arm A and 1.10 in Arm B (44.7%/40.5%) *ii*) the sample size is large (452 participants were enrolled) and a control arm is present [patients were randomized to one of two treatment arms: trastuzumab and capecitabine (Arm A, 224 participants) or pertuzumab with trastuzumab and capecitabine (Arm B, 228 participants)]; *iii*) the study population consist of patients with breast cancer, a solid tumour assessed by RECIST criteria. In the PHEREXA trial, the ORR was assessed using RECIST v1.0.^12^, ^14^ We carried out the present analysis, with two main objectives: first, to confirm at single‐patient level the discrepancy observed by analysing aggregated data; second, to investigate the factors leading to the discrepancies in the ORR assessment between LIs and BICR.

## 2 METHODS

### 2.1 Study design and patients

This is a secondary, independent, unplanned analysis of the outcome of patients included in the PHEREXA trial.^14^ In this trial, patients with HER2‐positive, metastatic breast cancer (MBC) were randomized 1:1 to receive either trastuzumab plus capecitabine or pertuzumab plus trastuzumab plus capecitabine. The primary outcome of the PHEREXA trial was independent review facility‐assessed (IRF) PFS. Secondary outcomes included: investigator‐assessed (INV) PFS, IRF‐ and INV‐assessed objective response rate (ORR). Details about the design and conduct of the PHEREXA trial were previously published elsewhwere.^14^ In this trial, tumour assessment was performed by using the Response Evaluation Criteria in Solid Tumours (RECIST) v1.0.^12^ Briefly, a first evaluation was performed at the screening and subsequently every 9 weeks from the random assignment until week 27 and every 12 weeks thereafter until the BICR confirmed disease progression.^14^ The Sponsor of the PHEREXA trial is Hoffmann‐La Roche. The Sponsor kindly made available individual patients' data for this analysis through the VIVLI platform. Hoffmann‐La Roche did not have any role into the design and conduction of this analysis.

Patients were eligible for this analysis if they displayed at least one “target” lesion at baseline, before starting the treatment. For each patient, we extracted data for: demographics (age, BMI, ethnicity, smoking status, baseline PS, Hormonal Receptor status, visceral/non‐visceral lesions); tumour assessment evaluations and definition of response (according to RECIST v1.0); reason for treatment discontinuation; group of target lesions.

### 2.2 Statistical analyses

The variables of interest were summarized through descriptive statistics, and the results were expressed as both absolute measures and proportions. Differences in proportions were assessed using Pearson's chi‐square test or Fisher's exact test. For each evaluation timepoint and each patient we calculated both investigator‐assessed (INV) and centrally independently reviewed (IRF) ORR, encompassing the Complete Response (CR) and the Partial Response (PR). Similarly, we also derived the Disease Control Rate (DCR), including also the Stable Disease (SD). All the response evaluations were made according to RECIST v1.0, consistently with the original PHEREXA design. All the analyses were performed both considering the entire population as a whole and within each treatment arm.

Discrepancy Index (DI) is defined as the ratio ORR(INV)/ORR(IRF) and is used as a measure of the expectation bias between LIs and BICR. Values >1 stand for a larger optimistic expectation by local investigators.

Agreement between local investigator and central evaluations was assessed by Cohen's κ.^15^ Although there is no universal definition of the interpretation of κ values, according to Fleiss,^16^ κ values < 0.40 can be interpreted as poor agreement, values between 0.40 and 0.75 as moderate to good agreement and values > 0.75 as excellent agreement.

To explore the factors potentially explaining the discrepancies between INV and IRF, we calculated Odds Ratio (OR) through both univariate and multivariate logistic models, having the ‘best response match’ as dependent variable and the organ of target lesion (brain, breast, liver, lung, lymph nodes, multiple organs and others); presence of target + non target *vs*. target only; number of target lesions (both continuously and divided in categories) and number of non‐target lesions (continuous), as covariate.

The aim of these analyses was exploratory, and there was no *a‐priori* hypothesis. A p‐value < 0.05 was considered statistically significant for all the tests. Due to the exploratory nature of the analysis, we did not correct for multiplicity.

All analyses were performed using R (R Foundation for Statistical Computing).

### 2.3 Nomenclature of targets and ligands

Key protein targets and ligands in this article are hyperlinked to corresponding entries in http://www.guidetopharmacology.org, and are permanently archived in the Concise Guide to PHARMACOLOGY 2021/22.^17^

## 3 RESULTS

PHEREXA was a multicentre, open‐label, randomized phase 3 study, aiming to test the efficacy of pertuzumab as an add‐on therapy to trastuzumab plus capecitabine (ClinicalTrials.gov identifier: NCT01026142). The study enrolled a total of 452 patients with demographic characteristics previously published.^14^ We gained access to the raw data from this clinical trial to perform an analysis at single‐patient level and investigate the reasons behind the discrepancy observed in the evaluation of the ORR between LIs and BICR.^10^ By applying the eligibility criteria of RECIST v1.0,^12^ we found 364 patients out of 452 (80.5%) with at least one target lesion at baseline according to the LI evaluation. These patients were considered eligible for the analysis. On the other hand, we defined 371 out of 450 (82.4%) subjects as eligible for the analysis using the BICR review dataset. In this dataset, data were not available for two patients. Demographic characteristics of the eligible participants according to the LI evaluation are presented in Table 1. Arm B included more patients of Asian origin (19.7% *vs*. 9.9%) and race (22.4% *vs*. 10%) compared with Arm A. A slightly lower number of patients with oestrogen‐ or progesterone‐receptor‐positive tumours was included in Arm B compared with Arm A.

**TABLE 1:** Demographic characteristics of the eligible participants according to the evaluation performed by local investigators.

Characteristics	Arm A (n = 181)	Arm B (n = 183)
Median age, years	55	54
Median BMI, kg/m^2^	25	25.9
World Region, No. (%)		
Asia	18 (9.9)	36 (19.7)
Europe	148 (81.8)	129 (70.5)
North America	3 (1.7)	6 (3.3)
South America	12 (6.6)	12 (6.6)
Race, No. (%)		
Asian	19 (10.5)	41 (22.4)
White	152 (84.0)	134 (73.2)
American Indian or Alaska native	1 (0.6)	1 (0.5)
Other	9 (5.0)	7 (3.8)
Smoking status, No. (%)		
Current smoker	13 (7.2)	21 (11.5)
Past smoker	26 (14.4)	24 (13.1)
Never smoked	142 (78.5)	136 (74.3)
Baseline ECOG PS, No. (%)		
PS 0	1185 (65.2)	126 (68.9)
PS 1	58 (32.0)	56 (30.6)
PS 2	2 (1.1)	1 (0.5)
Visceral disease lesions, No. (%)		
Visceral and non‐visceral lesions	101 (55.8)	109 (59.6)
Visceral lesions only	36 (19.9)	26 (14.2)
Oestrogen receptor status, No. (%)		
Positive	92 (50.8)	87 (47.5)
Negative	88 (48.6)	94 (51.4)
Unknown	1 (0.6)	2 (1.1)
Progesterone receptor status, No. (%)		
Positive	69 (38.1)	62 (33.9)
Negative	107 (59.1)	116 (63.4)
Unknown	5 (2.8)	5 (2.7)

We calculated the best response using the LI and BICR datasets (Supplementary Table 1). By analysing the data according to the LI on the eligible patients, we estimated an ORR (on the total number of eligible patients, n = 364) of 51.7% and a disease control rate (DCR) of 84.4% (Supplementary Table 2). In addition, we found an ORR of 45.3% and a DCR of 80.1% in Arm A, whereas the ORR was 57.9% with a DCR of 88.5% in Arm B. These results confirm the beneficial effects of pertuzumab in addition to standard therapy, according to the LI evaluation. We obtained similar results when we performed the analysis on all participants using the LI dataset (Supplementary Table 3). On the other hand, we calculated an ORR of 46.3% and DCR of 85.9% on the total number of eligible patients (n = 371) according to BICR (Supplementary Table 4). Moreover, we measured an ORR of 43.4% and DCR of 80.8% in Arm A; and ORR = 49.2% with DCR = 91.0% in Arm B. By applying the discrepancy index (DI = ORR by LI/ORR by BICR),^10^, ^11^ we confirmed the more optimistic evaluation by the LIs compared with the BICR review (ORR by LI 51.7%/ORR by BICR 46.3%, DI = 1.12 on the total number of assessments performed on the eligible patients by LIs and BICR).

To further analyse the level of agreement/disagreement among the two evaluations, we used a dichotomous variable, named best response match, with a value of ‘Yes’ if there was agreement between the two evaluations on the best response and ‘No’ in presence of disagreement between the local investigator (INV) and BICR (data are presented in Supplementary Table 6). On the total number of assessments, a Cohen's κ of 0.48, p < 0.001 was found, indicating moderate agreement between the two evaluations. A similar trend was observed by analysing each treatment arm separately: Cohen's κ (Arm A) = 0.54, p < 0.001; Cohen's κ (Arm B) = 0.43, p < 0.001. Similar results were obtained on the eligible patients by the LIs (Supplementary Table 7) and on the eligible patients by BICR (Supplementary Table 8). These results would therefore further confirm the modest agreement between the two assessments.

Additional analyses were carried out on the eligible patients according to the LI assessment to understand potential factors underlying the observed discrepancies. A summary of the tumour assessment details is provided in Table 2. We observed a higher number of partial and complete responses in Arm B compared with Arm A. This finding is consistent with improved outcomes associated with the addition of pertuzumab to standard therapy, as noted above. No relevant differences were observed between the two treatment groups with respect to the reasons of discontinuation and group of target lesions. However, it seems that the rate of best match was higher in Arm A compared with Arm B (Table 2). There were no significant associations between concordant and discordant evaluations in relation to the Group of target lesions, Target + non‐target lesions and the number of target and non‐target lesions analysed (Table 3).

**TABLE 2:** Details on tumour assessment by local investigators.

	Arm A (n = 181)	Arm B (n = 183)
Best response, No. (%)		
Unable to assess	10 (5.5)	2 (1.1)
Progressive disease	26 (14.4)	19 (10.4)
Stable disease	63 (34.8)	56 (30.6)
Partial response	77 (42.5)	95 (51.9)
Complete response	5 (2.8)	11 (6.0)
Reasons for discontinuation, No. (%)		
Admin/other	3 (1.7)	6 (3.3)
AE/Int. illness	18 (9.9)	13 (7.1)
Death	0	1 (0.5)
Insuff. therapy	143 (79.0)	153 (83.6)
Refused treatment	3 (1.7)	0
Viol. criteria	0	2 (1.1)
Withdrew	14 (7.7)	8 (4.4)
Group of target lesions, No. (%)		
Brain	1 (0.6)	5 (2.7)
Breast	11 (6.1)	8 (4.4)
Liver	46 (25.4)	43 (23.5)
Lung	20 (11.0)	28 (15.3)
Lymph nodes	18 (9.9)	26 (14.2)
Lymph nodes + Organ	35 (19.3)	40 (21.9)
Multiple organs	37 (20.4)	23 (12.6)
Other	13 (7.2)	10 (5.5)
Best response match, No. (%)		
Yes	132 (72.9)	116 (63.4)
No	49 (27.1)	67 (36.6)

**TABLE 3:** Distribution of “best response match” status across variable of interest.

Best response match	Yes (n = 248)	No (n = 116)
Group of target lesions, No. (%)		
Brain	3 (50.0)	3 (50.0)
Breast	10 (52.6)	9 (47.4)
Liver	65 (73.0)	24 (27.0)
Lung	31 (64.6)	17 (35.4)
Lymph nodes	26 (59.1)	18 (40.9)
Lymph nodes + organ	58 (77.3)	17 (22.7)
Multiple organs	43 (71.7)	17 (28.3)
Other	12 (52.2)	11 (47.8)
Target + non target disease, No. (%)		
Yes	50 (73.5)	18 (26.5)
No	198 (66.9)	98 (33.1)
Number of target lesions, median	2	2
Number of non‐target lesions, median	2	1.5

We performed univariate analysis and multivariate logistic regression analysis using the best match as the response (dependent) variable to further analyse the reasons behind the observed discrepancy. At the univariate analysis, only isolated lymph nodes yielded a statistically significant association with the discrepancy of evaluation (OR = 2.36; 95% CI = 1.05–5.30, p = 0.037) (Supplementary Table 9). This was confirmed by multivariate analysis (Table 4). In addition, the number of target lesions was a significant factor contributing to the discrepancy between the two evaluations (Table 4). The probability of discrepancy increased by 20% with the increasing number of target lesions evaluated. In the categorical model, the selection of 8–10 lesions for the evaluation was associated with a five‐fold higher mean risk of discrepancy (CI 95% = 1.21–24.92, p < 0.03) (Table 5). Finally, in comparison to the analysis of lymph nodes + organ, the evaluation of breast, lung and lymph nodes (only) significantly increases the probability of discrepancy between the two evaluations (Tables 4 and 5). As shown in Table 5, the likelihood of discrepancy between LIs and BICR significantly increased for the evaluation of breast lesions (OR = 4.05, 95% CI = 1.30–12.78, p = 0.02), lung lesions (OR = 2.60, 95% CI = 1.06–6.54, p = 0.04) and lymph nodes (only) (OR = 3.02; 95% CI = 1.24–7.55, p < 0.02) in comparison to the evaluation of lymph nodes + organ.

**TABLE 4:** Results from the multivariate logistic regression model. The response variable (best response match) is coded as 1 = ‘no’, 0 = ‘yes’. For the analysis of target lesions the reference was set as the lymph nodes + organ.

	OR (95% CI)	p value
Group of target lesions		
Lymph nodes + organ (Ref.)		
Breast	5.14 (1.57–17.19)	**0.01**
Liver	1.91 (0.84–4.47)	0.13
Lung	3.11 (1.22–8.11)	**0.02**
Lymph nodes	3.42 (1.39–8.71)	**0.01**
Brain	4.71 (0.77–29.22)	0.08
Multiple organs	1.46 (0.65–3.31)	0.36
Other	5.54 (1.80–17.50)	**0.003**
Target + non target disease		
No (Ref.)		
Yes	1.56 (0.79–3.16)	0.21
Number of target lesions	1.20 (1.02–1.41)	**0.02**
Number of non target lesions	0.97 (0.85–1.09)	0.65

**TABLE 5:** Results from the multivariate logistic regression model. The response variable (best response match) is coded as 1 = ‘no’, 0 = ‘yes’. Number of target lesions is coded as a categorical variable.

	OR (95% CI)	p value
Group of target lesions		
Lymph nodes + organ (Ref.)		
Breast	4.05 (1.30–12.78)	**0.02**
Liver	1.68 (0.76–3.86)	0.21
Lung	2.60 (1.06–6.54)	**0.04**
Lymph nodes	3.02 (1.24–7.55)	**0.02**
Brain	4.26 (0.71–25.94)	0.10
Multiple organs	1.52 (0.67–3.51)	0.32
Other	4.35 (1.49–13.02)	**0.01**
Target + non target disease		
No (Ref.)		
Yes	1.48 (0.75–3.00)	0.27
Number of non target lesions	0.97 (0.85–1.09)	0.59
Number of target lesions		
1–3 (Ref.)		
4–7	1.39 (0.72–2.66)	0.3
8–10	5.27 (1.21–24.92)	**0.03**

## 4 DISCUSSION

In the present paper, we included the results of the re‐analysis of the data generated during the PHEREXA trial.^14^ The analysis was carried out at the single patient level by re‐assessing the evaluations of the disease status performed by LIs and BICR during the trial. By applying the RECIST v1.0 criteria used in the trial, we considered eligible for the analyses patients with at least 1 target lesion at the screening evaluation. We found 364/452 eligible subjects by LIs and 371/450 eligible by BICR. These data suggest potential inter‐reader variability in the selection of target lesions at baseline. In fact, the selection of target lesions at baseline or their measure throughout the trial may vary between readers^18^ with a significant impact on subsequent assessment of tumour response. Another source of variability can be the quantitative evaluation of non‐target lesions and the detection of new lesions,^18^ other factors that may contribute to discrepancies observed in the ORR evaluation. The latter provides an initial evaluation of treatment efficacy, based on the hypothesis that a decrease in tumour size would correlate with survival benefits.^19^ Notably, an increasing number of drugs have been approved, either through accelerated or regular approval, by the FDA based on the evaluation of the ORR in Phase 1–2 clinical trials,^20^ despite the poor correlation between ORR and overall survival and PFS.^21^

The main objective of the evaluation of radiological images to assess tumour response to treatment is to categorize patients into four groups, i.e., complete response (CR), partial response (PR), stable disease (SD) and progressive disease (PD).^19^ These categories are then used to estimate the best overall response (BOR) and the ORR. As reported in the supplementary material, we used these categorizations to first confirm the observation that LIs were more optimistic in the evaluation of the ORR compared with the central reviewers. We achieved this result by applying the DI previously used in our analyses.^10^, ^11^ In addition, we found a Cohen's κ of 0.48 (p < 0.001) between the two evaluations on the total number of assessments, indicating moderate agreement between LIs and BICR. Importantly, the probability of discrepancy increased by 20% with the increasing number of target lesions evaluated. Therefore, the selection of a measurable disease is a crucial starting point for the correct assessment of the ORR. In line with these observations, we found that the number of target lesions was a major determinant of discrepancy between the two evaluations. The selection of 8–10 lesions for the evaluation was associated with a 5‐fold higher mean risk of discrepancy (CI 95% = 1.21–24.92, p < 0.03).

Taken together, these results highlight the issue of the inter‐reader variability as a potential factor underlying the discrepancy observed between the evaluations of radiological images at the local level compared with central reviews. A retrospective analysis carried out on five BICR clinical trials requiring double central reads in the lung cancer setting showed, among others, a discrepancy rate of 28.8% related to the evaluation of the BOR between two central readers.^22^ In these trials, there were often discrepancies related to the selection of different target and non‐target lesions among readers, as well as the possibility that one reader did not identify any measurable disease in certain patients. Consistently, we found a different number of eligible patients for the re‐analysis using the two sets of evaluations provided within the trial.

In addition, inter‐reader reproducibility seems to be lower for the evaluation of lesions in specific sites such as lymph nodes and peritoneal disease.^23^ In this context, we found that the likelihood of discrepancy between LIs and BICR significantly increased for the evaluation of breast and lung lesions and lymph nodes (only). These data could be, at least hypothetically, explained by inherent radiological and methodological factors. Breast lesions mandated no specific minimum size criterion in RECIST v1.0, in contrast to the specific thresholds for other sites (solid organs ≥10 mm, lymph nodes ≥15 mm short axis). This lack of standardization could have increased interpretative heterogeneity. Moreover, information about breast lesions could come from different radiological (i.e. mammography, US) and clinical sources (i.e. palpation) that can induce “fusion‐bias” in local investigators, being more prone to evaluate the different sources in the response assessment. In this perspective, the accuracy of breast lesion measurements demonstrates substantial modality‐dependent variability: concordance with pathological specimen measurement ranges from 64.3% (mammography) to 82.1% (MRI), and the absence of mandated imaging protocols could introduce systematic measurement divergence between local and central assessors.^24^ Pulmonary nodules in the 10–15 mm range could represent a measurement ‘grey zone’ where inter‐observer CT variability reaches 4.5–5.3%, rendering categorical response distinctions vulnerable to observer‐dependent interpretation. Additionally, RECIST v1.0 lacked the absolute 5 mm progression threshold introduced in v1.1, further amplifying this vulnerability. Lymph nodes as isolated target lesions present distinct challenges: target selection variability increases linearly with available lesion number, and coalescence/fragmentation phenomena are inadequately captured by unidimensional RECIST measurement.^25^

In our previous studies, we found a significant overestimation of the ORR by the LIs compared with BICR both in phase 2 and phase 3 trials. This effect was more relevant in uncontrolled clinical trials, i.e., trials lacking a comparator arm.^10^, ^11^ In contrast, a recent meta‐analysis carried out on early phase (phase 1/2 and 2) clinical trials found a greater agreement between the two methods of evaluation.^26^ The meta‐analysis included 20 trials that led to drug approval between 1 January 2020 and 30 June 2024. The trials were all open‐label and lacked a comparator arm. The analysis comprised 31 paired evaluations, including also subgroup assessments. The LIs were found more optimistic than BICR in 15/31 evaluations, whereas BICR was found more optimistic than LIs in 14/31 evaluations, and in two cases, no differences were reported. The pooled analysis showed no significant difference between the two evaluation methods (OR = 0.98, 95% CI: 0.87–1.11, p = 0.75 and *I* ^*2*^ = 0%). It is possible that better compliance with the RECIST criteria and knowledge of the guidelines can reduce inter‐reader variability in modern trials.^18^ At variance of the PHEREXA trial,^14^ all studies included in this meta‐analysis assessed the ORR by RECIST v1.1.^13^ One major difference between RECIST v1.0 and v1.1. is related to the number of target lesions allowed at the baseline for eligibility. That is up to a total of 10 target lesions, maximum 5 per organ, in RECIST v1.0.^12^ The number was reduced to a total of 5 target lesions, maximum two per organ, in RECIST v1.1.^13^ This is consistent with our findings that the probability of discrepancy increases with the number of target lesions selected at baseline. On the other hand, it has been shown that the inclusion of the same (sub)group of patients, multiple times, in the same meta‐analysis may contribute to the inflation of the results.^27^ In this context, our studies cannot be formally considered as meta‐analyses but rather comparative studies where each trial was taken as the experimental unit, and the analysis was carried out on paired data generated from each endpoint, with data provided by BICR being taken as the control.^10^, ^11^ Differences in the methodology adopted may explain the different outcome of this meta‐analysis in comparison to our studies. Notably, a recent uncontrolled phase 2 trial (ClinicalTrials.gov identifier: NCT03807778), with an open‐label design, carried out in Japanese patients with locally advanced or metastatic non‐small cell lung cancer and testing the efficacy of mobocertinib, seems to confirm the trend towards an overestimation of the ORR by LI.^28^ Briefly, the estimation of the ORR by BICR in the full analysis set (FAS) population was 18.2% (6/33, 95% CI 7.0–35.5) whereas the ORR by LIs was 39.4% (13/33, 95% CI 22.9–57.9). This would lead to a DI of 2.16. Notably, in this trial, the RECIST v1.1 criteria were adopted. This may also depend on the contextual knowledge bias between the two reviewers, considering that during central analysis, the radiologists are often blinded to clinical information and historical examinations.^18^ Interestingly, in this trial, while the central reviewer classified one patient out of 6 responders (3.0%) with a CR as BOR, the LIs claimed all PRs. This further supports the notion that the kind of discrepancy between the two evaluations may vary with the clinical outcome and during the trial execution.^22^

We can conclude that addressing ORR in cancer clinical trials is still a substantially subjective process. Choices like: selection of target lesions at baseline, measurement of the lesions, as well as imaging technological availability, effectively affect the proper application of rules and guidelines like RECIST. This is unarguably true with the increasing number of target lesions and particularly difficult districts (i.e. lymph nodes and peritoneum). As mentioned above, in the revised version of RECIST criteria v1.1, the number of target lesion was reduced from 10 to only 5 in total and nodes criteria evolved with the improvement of technological capability, already pictured it in 2009.^13^ Therefore, the overestimation of the tumour response by LIs may be related to the intrinsic variability of the application of the RECIST criteria. In case of early phase clinical trials, in which it is important to determine the extent of the effect in order to inform future drug development, our studies support the adoption of the two evaluations. Given the potential for LIs to overestimate the benefits of a novel drug, confirmation at the central level is important. BICR assessments can strengthen the results obtained locally and support further clinical development.

## AUTHOR CONTRIBUTIONS

G.D.: analysing, revising and editing; T.G.: analysing; P.N.: conceptualization, supervision of the entire project, revising and editing; C.D.R.: conceptualization, writing of the original draft.

## CONFLICT OF INTEREST STATEMENT

The authors declared no competing interests for this work.

## Supporting information


**Table S1.** Best response by local investigators (INV) and according to the independent review facility‐assessed (IRF).
**Table S2.** Best response by INV (eligible patients).
**Table S3.** Best response by INV (all patients).
**Table S4.** Best response by IRF (eligible patients).
**Table S5.** Best response by IRF (all patients).
**Table S6.** Best response match (all patients).
**Table S7.** Best response match (eligible patients according to INV review).
**Table S8.** Best response match (eligible patients according to IRF review).
**Table S9.** Results from the univariate analysis.

## Data Availability

The data that support the findings of this study are available through Vivli, Inc.

## References

[1] Chvetzoff G , Tannock IF . Placebo effects in oncology. J Natl Cancer Inst. 2003;95:19‐29. doi:10.1093/jnci/95.1.19 12509397

[2] Chaugule PD , Varpe PC , Tandulje AA , Raghuvanshi RS , Srivastava S . Expedited pathway insights: unveiling oncology and non‐oncology drug approvals and withdrawals of USFDA and EMA. Crit Rev Oncol Hematol. 2025;205:104539. doi:10.1016/j.critrevonc.2024.104539 39521307

[3] Ranganathan S , Prasad V , Olivier T . The fate of sotorasib: a regulatory failure potentially harming patients. Lancet Oncol. 2024;25(5):549‐552. doi:10.1016/S1470-2045(23)00616-2 38697153

[4] Clinical Trial Endpoints for the Approval of Cancer Drugs and Biologics Guidance for Industry December 2018. The US FDA website. Accessed July 16, 2025. https://www.fda.gov/regulatory-information/search-fda-guidance-documents/clinical-trial-endpoints-approval-cancer-drugs-and-biologics

[5] Evaluation of anticancer medicinal products ‐ Scientific guideline (V5). The EMA website. Accessed July 16, 2025. https://www.ema.europa.eu/en/evaluation-anticancer-medicinal-products-scientific-guideline#current-version-revision-7-8971

[6] Dodd LE , Korn EL , Freidlin B , et al. Blinded independent central review of progression‐free survival in phase III clinical trials: important design element or unnecessary expense? J Clin Oncol. 2008;26(22):3791‐3796. doi:10.1200/JCO.2008.16.1711 18669467 PMC2654812

[7] Amit O , Mannino F , Stone AM , et al. Blinded independent central review of progression in cancer clinical trials: results from a meta‐analysis. Eur J Cancer. 2011;47(12):1772‐1778. doi:10.1016/j.ejca.2011.02.013 21429737

[8] Dello Russo C , Navarra P . What is the weight of expectation bias in oncology trials? Br J Clin Pharmacol. 2023;89:1279‐1281. doi:10.1111/bcp.15680 36772876

[9] Dello Russo C , Cappoli N , Navarra P . A comparison between the assessments of progression‐free survival by local investigators versus blinded independent central reviews in phase III oncology trials. Eur J Clin Pharmacol. 2020;76(8):1083‐1092. doi:10.1007/s00228-020-02895-z 32447437

[10] Dello Russo C , Navarra P . Local investigators significantly overestimate overall response rates compared to blinded independent central reviews in uncontrolled oncology trials: a comprehensive review of the literature. Front Pharmacol. 2022;13:858354. doi:10.3389/fphar.2022.858354 35652050 PMC9149259

[11] Dello Russo C , Cappoli N , Pilunni D , Navarra P . Local investigators significantly overestimate overall response rates compared to blinded independent central reviews in phase 2 oncology trials. J Clin Pharmacol. 2021;61(6):810‐819. doi:10.1002/jcph.1790 33244770

[12] Therasse P , Arbuck SG , Eisenhauer EA , et al. New guidelines to evaluate the response to treatment in solid tumors. European Organization for Research and Treatment of cancer, National Cancer Institute of the United States, National Cancer Institute of Canada. J Natl Cancer Inst. 2000;92(3):205‐216. doi:10.1093/jnci/92.3.205 10655437

[13] Eisenhauer EA , Therasse P , Bogaerts J , et al. New response evaluation criteria in solid tumours: revised RECIST guideline (version 1.1). Eur J Cancer. 2009;45(2):228‐247. doi:10.1016/j.ejca.2008.10.026 19097774

[14] Urruticoechea A , Rizwanullah M , Im SA , et al. Randomized phase III trial of trastuzumab plus capecitabine with or without Pertuzumab in patients with human epidermal growth factor receptor 2‐positive metastatic breast cancer who experienced disease progression during or after trastuzumab‐based therapy. J Clin Oncol. 2017;35:3030‐3038. doi:10.1200/JCO.2016.70.6267 28437161

[15] Cohen J . A coefficient of agreement for nominal scales. Educ Psychol Meas. 1960;20(1):37‐46. doi:10.1177/001316446002000104

[16] Fleiss JL . Statistical methods for rates and proportions. 2nd ed. John Wiley & Sons; 1981:38‐46.

[17] Alexander SP , Kelly E , Mathie A , et al. THE CONCISE GUIDE TO PHARMACOLOGY 2021/22: Introduction and other protein targets. Br J Pharmacol. 2021;178(Suppl 1):S1‐S26. doi:10.1111/bph.15537 34529830 PMC9513948

[18] Iannessi A , Beaumont H , Ojango C , Bertrand AS , Liu Y . RECIST 1.1 assessments variability: a systematic pictorial review of blinded double reads. Insights Imaging. 2024;15(1):199. doi:10.1186/s13244-024-01774-w 39112819 PMC11306910

[19] Aykan NF , Özatlı T . Objective response rate assessment in oncology: current situation and future expectations. World J Clin Oncol. 2020;11(2):53‐73. doi:10.5306/wjco.v11.i2.53 32133275 PMC7046919

[20] Chen EY , Raghunathan V , Prasad V . An overview of cancer drugs approved by the US Food and Drug Administration based on the surrogate end point of response rate. JAMA Intern Med. 2019;179(7):915‐921. doi:10.1001/jamainternmed.2019.0583 31135822 PMC6547222

[21] Cooper K , Tappenden P , Cantrell A , Ennis K . A systematic review of meta‐analyses assessing the validity of tumour response endpoints as surrogates for progression‐free or overall survival in cancer. Br J Cancer. 2020;123(11):1686‐1696. doi:10.1038/s41416-020-01050-w 32913287 PMC7687906

[22] Beaumont H , Iannessi A . Can we predict discordant RECIST 1.1 evaluations in double read clinical trials? *Front* . Oncologia. 2023;13:1239570. doi:10.3389/fonc.2023.1239570 PMC10585359 37869080

[23] Krasovitsky M , Lee YC , Sim HW , et al. Interobserver and intraobserver variability of RECIST assessment in ovarian cancer. Int J Gynecol Cancer. 2022;32(5):656‐661. doi:10.1136/ijgc-2021-003319 35379690

[24] Azhdeh S , Kaviani A , Sadighi N , Rahmani M . Accurate estimation of breast tumor size: a comparison between ultrasonography, mammography, magnetic resonance imaging, and associated contributing factors. Eur J Breast Health. 2020;17(1):53‐61. doi:10.4274/ejbh.2020.5888 33796831 PMC8006785

[25] Muenzel D , Engels HP , Bruegel M , Kehl V , Rummeny EJ , Metz S . Intra‐ and inter‐observer variability in measurement of target lesions: implications for response assessment in oncology. Radiology. 2012;266:946‐954. 10.2478/v10019-012-0009-z PMC3423763 22933974

[26] Zettler ME . Assessment of objective response rate by investigator vs. blinded independent central review in pivotal trials of oncology drugs for solid tumor indications. Cancer. 2025;17(7):1096. doi:10.3390/cancers17071096 PMC11987735 40227586

[27] Kanellopoulou A , Dwan K , Richardson R . Common statistical errors in systematic reviews: a tutorial. Cochrane Evid Synth Methods. 2025;3(2):e70013. doi:10.1002/cesm.70013 40475918 PMC11795887

[28] Yoh K , Azuma K , Hayashi H , et al. A phase 2 study of mobocertinib as first‐line treatment in Japanese patients with non‐small cell lung cancer harboring EGFR exon 20 insertion mutations. Int J Clin Oncol. 2024;29(10):1461‐1474. doi:10.1007/s10147-024-02588-y 39190099 PMC11420270

