# Supplementary material for: Main factors associated with variability in the assessment of the objective response rate. A re‐analysis of the PHEREXA phase 3 clinical trial
Source: Br J Clin Pharmacol. 2026 Jan 31;92(6):1894–901. doi: 10.1002/bcp.70464 (PMC13206187; doi:10.1002/bcp.70464)
Supplement: Supplementary file 1 — Table S1. Best response by local investigators (INV) and according to the independent review facility‐assessed (IRF). Table S2. Best response by INV (eligible patients). Table S3. Best response by INV (all patients). Table S4. Best response by IRF (eligible patients). Table S5. Best response by IRF (all patients). Table S6. Best response match (all patients). Table S7. Best response match (eligible patients according to INV review). Table S8. Best response match (eligible patients according to IRF review). Table S9. Results from the univariate analysis. [file BCP-92-1894-s001.docx]

**Supplementary Tables**

**Supplementary Table 1. Best response by local investigators (INV) and according to the independent review facility-assessed (IRF).**

|  | **INV review, n (%)** | **IRF review, n (%)** |
| --- | --- | --- |
| **Unable to assess** | 14 (3.1) | 16 (3.6) |
| **Progressive disease** | 53 (11.7) | 43 (9.6) |
| **Stable disease** | 186 (41.2) | 215 (47.8) |
| **Partial response** | 177 (39.2) | 166 (36.9) |
| **Complete response** | 22 (4.9) | 10 (2.2) |

**Supplementary Table 2. Best response by INV (eligible patients)**

|  | **Arm A, n = 181 (%)** | **Arm B, n = 183 (%)** | **Total, n = 364** |
| --- | --- | --- | --- |
| **Unable to assess** | 10 (5.5) | 2 (1.1) | 12 (3.3) |
| **Progressive disease** | 26 (14.4) | 19 (10.4) | 45 (12.4) |
| **Stable disease** | 63 (34.8) | 56 (30.6) | 119 (32.7) |
| **Partial response** | 77 (42.5) | 95 (51.9) | 172 (47.3) |
| **Complete response** | 5 (2.8) | 11 (6.0) | 16 (4.4) |

**Supplementary Table 3. Best response by INV (all patients)**

|  | **Arm A, n = 224 (%)** | **Arm B, n = 228 (%)** | **Total, n = 452** |
| --- | --- | --- | --- |
| **Unable to assess** | 10 (4.5) | 4 (1.8) | 14 (3.1) |
| **Progressive disease** | 31 (13.8) | 22 (9.6) | 53 (11.7) |
| **Stable disease** | 93 (41.5) | 93 (40.8) | 186 (41.2) |
| **Partial response** | 81 (36.2) | 96 (42.1) | 177 (39.2) |
| **Complete response** | 9 (4.0) | 13 (5.7) | 22 (4.9) |

**Supplementary Table 4. Best response by IRF (eligible patients)**

|  | **Arm A, n = 182 (%)** | **Arm B, n = 189 (%)** | **Total, n = 371** |
| --- | --- | --- | --- |
| **Unable to assess** | 10 (5.5) | 4 (2.1) | 14 (3.8) |
| **Progressive disease** | 25 (13.7) | 13 (6.9) | 38 (10.2) |
| **Stable disease** | 68 (37.4) | 79 (41.8) | 147 (39.6) |
| **Partial response** | 79 (43.4) | 87 (46.0) | 166 (44.7) |
| **Complete response** | 0 | 6 (3.2) | 6 (1.6) |

**Supplementary Table 5. Best response by IRF (all patients)**

|  | **Arm A, n = 222 (%)** | **Arm B, n = 228 (%)** | **Total, n = 450** |
| --- | --- | --- | --- |
| **Unable to assess** | 11 (4.95) | 5 (2.2) | 16 (3.6) |
| **Progressive disease** | 26 (11.71) | 17 (7.5) | 43 (9.6) |
| **Stable disease** | 105 (47.3) | 110 (48.2) | 215 (47.8) |
| **Partial response** | 79 (35.59) | 87 (38.2) | 166 (36.9) |
| **Complete response** | 1 (0.45) | 9 (3.9) | 10 (2.2) |

**Supplementary Table 6. Best response match (all patients)**

|  | **Arm A, n (%)** | **Arm B, n (%)** | **Total, n (%)** |
| --- | --- | --- | --- |
| **Yes** | 156 (69.6) | 145 (63.6) | 301 (66.6) |
| **No** | 68 (30.4) | 83 (36.4) | 151 (33.4) |

**Supplementary Table 7. Best response match (eligible patients according to INV review)**

|  | **Arm A, n (%)** | **Arm B, n (%)** | **Total, n (%)** |
| --- | --- | --- | --- |
| **Yes** | 132 (72.9) | 116 (63.4) | 248 (68.1) |
| **No** | 49 (27.1) | 67 (36.6) | 116 (31.9) |

**Supplementary Table 8. Best response match (eligible patients according to IRF review)**

|  | **Arm A, n (%)** | **Arm B, n (%)** | **Total, n (%)** |
| --- | --- | --- | --- |
| **Yes** | 130 (71.4) | 123 (65.1) | 253 (67.6) |
| **No** | 52 (28.6) | 66 (34.9) | 118 (31.6) |

**Supplementary Table 9. Results from the univariate analysis.**

| **Category** | **OR (95% CI)** | **p value** |
| --- | --- | --- |
| Breast | 3.07 (1.07-8.78) | 0.115 |
| Lung | 1.87 (0.84-4.17) | 0.179 |
| Liver | 1.26 (0.62-2.57) | 0.529 |
| Lymph nodes (only) | 2.36 (1.05-5.30) | **0.037** |
| Multiple organs | 1.26 (0.60-2.64) | 0.548 |
| Other | 2.79 (1.01-7.70) | 0.059 |
